# Emphysematous prostatitis in renal transplant

**DOI:** 10.4103/0970-1591.36728

**Published:** 2007

**Authors:** Krishnaswamy Sampathkumar, Tanjore R. Murali, Yesudas S. Sooraj, Amol R. Mahaldar

**Affiliations:** Department of Nephrology, Meenakshi Mission Hospital and Research Centre, Lake Area, Melur Road, Madurai - 625 107, India; * Department of Urology, Meenakshi Mission Hospital and Research Centre, Lake Area, Melur Road, Madurai - 625 107, India

**Keywords:** Diabetes, emphysematous prostatitis, multiorgan failure, renal transplant, septic shock, septicemia, urinary tract infection

## Abstract

Urinary tract infections are common following renal transplant. The spectrum varies from asymptomatic bacteriuria to septicemia. Gas-producing infections of the urinary tract are rare but tend to have a grave prognosis when they do occur. We report a 57-year-old gentleman who underwent a renal transplant 20 months earlier. He presented to us with fever and dysuria. Clinical examination revealed a febrile and ill-looking patient with severe graft tenderness. An emergency pelvic CT scan revealed presence of emphysematous prostatitis, cystitis and pyelitis. Urine and blood cultures grew *E. coli*. Endoscopic abscess drainage was done and antibiotics given but he succumbed to his illness due to multiorgan failure within 48h. This is the first reported case of emphysematous prostatitis in a renal allograft recipient.

## INTRODUCTION

Urinary tract infection is the most common cause of sepsis in the post renal transplant period. It occurs in about 25% of renal transplant recipients of whom 33% develop acute pyelonephritis.[1] Multiple risk factors are responsible for this high incidence such as immunosuppressed state, diabetes, antibiotic use and instrumentations. Gas-producing infections account for a very small percentage of these infections but they are very important because of their life-threatening potential. So far only 12 cases of emphysematous pyelonephritis have been described in renal transplants and no case of emphysematous prostatitis has been described in the literature.[2] This is the first case of emphysematous prostatitis to be reported in a renal allograft recipient.

## CASE REPORT

A 57-year-old gentleman, who had undergone renal transplantation 20 months back presented with history of cloudy urine, chills and fever with pain over the renal allograft of two days duration. He was a known diabetic for 23 years, a known hypertensive for five years and was diagnosed as having end stage renal disease two years back. He was initiated on continuous ambulatory peritoneal dialysis and he underwent live unrelated renal transplantation eight months later. Pre-transplant cystoscopic evaluation had revealed Grade I occlusive lateral lobes of the prostate. MCU had revealed a bladder capacity of 450 ml without post void residual urine. He was given maintenance immunosuppression with tacrolimus, mycophenolate mofetil and prednisolone. One and a half months earlier, he was treated for acute rejection with pulse methyl prednisolone. He had also complained of excessive frequency of urination at night along with incomplete sense of bladder evacuation for the past one month. Per-rectal examination had revealed Grade 3 enlarged prostate. The serum Prostate Specific Antigen was normal. Foley's catheter was inserted and 200ml of urine was drained. Urinalysis showed only one to two pus cells, blood sugar was 181mg% and the urine culture grew *E. coli* sensitive to ceftriaxone. Ultrasonogram revealed an enlarged prostate. He was treated with intravenous ceftriaxone, sulbactum combination for 10 days. He refused to undergo endoscopic examination of his urinary tract. Dutasteride and tamsulosin were prescribed. Foley's catheter was removed after two weeks and trial voiding was successful. His PVRU at the time was 10ml. He declined transurethral prostatectomy which was offered to him during this time. He did not come for follow-up in the interim period.

**Figure 1:**
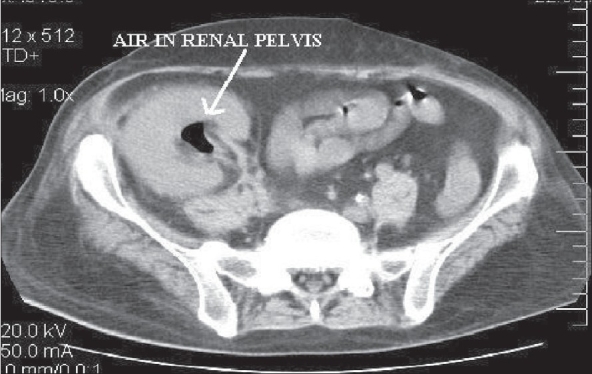
Emphysematous pyelitis

**Figure 2:**
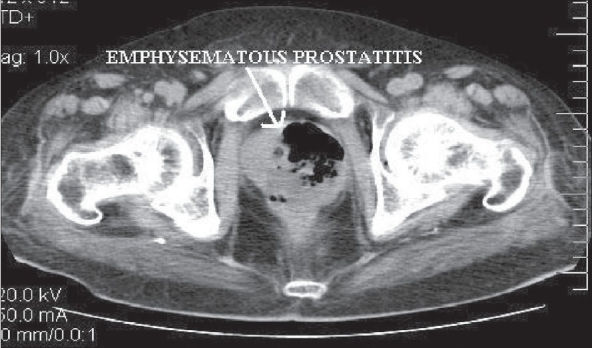
Emphysematous prostatitis

Clinical examination revealed a toxic-looking gentleman who was pale and icteric. He denied voiding symptoms this time. He was febrile (temperature was 100°F), his pulse rate was 101/min and his blood pressure was 160/100 mmHg. Abdominal examination revealed severe tenderness over the renal allograft. Rectal examination was not performed. Initial investigations showed hemoglobin of 10.2 gm/dl and a total count of 9,900 cells/cu.mm (polymorphs - 60%, Lymphocytes - 37%, eosinophils - 3%). His blood sugar was 101 mg%, blood urea was 137 mg/dl and serum creatinine was 3.1 mg/dl. Ultrasonogram of the renal allograft revealed an enlarged kidney with multiple airpockets within the collecting system. Doppler studies showed increased diastolic flow resistance of segmental arteries with resistive index of 0.78. A CT scan of the abdomen and pelvis revealed an enlarged allograft with presence of gas in the collecting system [Figure 1] Gas was also demonstrated in the bladder and prostate was enlarged with air pockets [Figure 2]. A diagnosis of emphysematous prosatitis, pyelitis and cystitis was made.

Foley's catheterization was done and it drained frank pus. He was started on imipenem/cilastatin. Emergency urology consult was obtained and cystourethroscopic prostatic abscess drainage was carried out within 4h of admission. Percutaneous lower calyceal puncture was done under ultrasound guidance and anterograde ‘Double J’ stenting of renal allograft was carried out. However, his renal parameters worsened and he was started on continuous arteriovenous hemofiltration. He developed septic shock and respiratory failure requiring mechanical ventilation. Meanwhile his urine culture grew *E. coli* sensitive to imipenem/cilastain which he was already receiving. Later blood culture also grew *E. coli*. In spite of the above measures he died within 48h of admission due to multiorgan failure.

## DISCUSSION

Our patient had several factors which could have contributed to this fulminant infection, namely diabetes mellitus, anti-rejection therapy and prior catheterization of urinary tract in the post transplant setting. In retrospect, during the previous admission if transurethral resection had been carried out the complication could have been averted. The case highlights the importance of close follow-up and aggressive management of patients in the post transplant period with urinary tract infections.

Emphysematous pyelonephritis is a gas-producing, necrotizing infection involving the renal parenchyma and perirenal tissue. More than 90% of cases occur in diabetics. Diabetics may also develop emphysematous pyelitis (i.e. gas in the renal pelvis) or cystitis with or without associated emphysematous pyelonephritis. Urinary tract obstruction is also a predisposing factor as in our case. Gas-forming urinary tract infections are usually associated with diabetes mellitus and obstruction of the collecting system. Bacteria such as *E. coli* are facultative anaerobes which can ferment glucose and fructose to produce carbon dioxide and hydrogen. The gas then becomes entrapped by an obstructive process.[3] Depending on the site of gas collection in the upper urinary tract the treatment varies from percutaneous drainage to bilateral nephrectomy. The mortality is high (20%) mainly because of the failure to diagnose the condition before generalized sepsis and multiorgan failure has occurred.[3]

Emphysematous prostatitis is an extremely rare condition with only a few cases having been reported in the literature. So far there have not been any case reports in renal transplant recipients. The reported etiologic microorganisms include *Klebsilla pneumoniae, Pseudomonas aeruginosa, Bacteroides fragilis* and *Candida albicans*.[4,5] *E. coli* has not so far been reported in the literature. The presenting symptoms of emphysematous prostatitis are nonspecific and usually they are treated as having a simple urinary tract infection. Transrectal ultrasound and CT scan should be performed on all patients in whom emphysematous prostatitis is suspected. Procedures of pus drainage in prostatic abscess include transurethral drainage, transurethral incision, perineal incision and transperineal prostatic puncture.[4] Transurethral drainage is the ideal method with minimal risk of bacteremia or sepsis.[4] However the mortality still remains high.

We report for the first time in the literature, a case of emphysematous prostatitis in a post renal transplant setting. We hereby highlight the underlying risk factors and clinical picture of such a rare manifestation. Prompt diagnosis and aggressive medical and surgical management are crucial. Mortality remains high.
